# Cardiovascular autonomic nervous system responses and orthostatic intolerance in astronauts and their relevance in daily medicine

**DOI:** 10.1007/s10072-022-05963-7

**Published:** 2022-02-23

**Authors:** Jens Jordan, Ulrich Limper, Jens Tank

**Affiliations:** 1 Institute of Aerospace Medicine, German Aerospace Center DLR, Linder Hoehe, 51147 Cologne, Germany; 2 Aerospace Medicine, Medical Faculty, University of Cologne, Cologne, Germany; 3 Department of Anesthesiology and Intensive Care Medicine, Merheim Medical Center, Hospitals of Cologne, University of Witten/Herdecke, Cologne, Germany

**Keywords:** Autonomic nervous system, Cardiovascular reflexes, Baroreflex, Orthostatic tolerance, Orthostatic hypotension, Space flight

## Abstract

**Background:**

The harsh environmental conditions during space travel, particularly weightlessness, impose a major burden on the human body including the cardiovascular system. Given its importance in adjusting the cardiovascular system to environmental challenges, the autonomic nervous system has been in the focus of scientists and clinicians involved in human space flight. This review provides an overview on human autonomic research under real and simulated space conditions with a focus on orthostatic intolerance.

**Methods:**

The authors conducted a targeted literature search using Pubmed.

**Results:**

Overall, 120 articles were identified and included in the review.

**Conclusions:**

Postflight orthostatic intolerance is commonly observed in astronauts and could pose major risks when landing on another celestial body. The phenomenon likely results from changes in volume status and adaptation of the autonomic nervous system to weightlessness. Over the years, various non-pharmacological and pharmacological countermeasures have been investigated. In addition to enabling safe human space flight, this research may have implications for patients with disorders affecting cardiovascular autonomic control on Earth.

## Introduction

Following the first human space flight in the early 1960s of the last century by cosmonaut Yuri Gagarin, human space programs evolved rapidly. In 1969, the astronaut Neil Armstrong was the first human being to set foot on the Moon. Subsequently the American Space Shuttle program was launched, and the Russian MIR space station was established. Perhaps, the most astonishing accomplishment in recent decades was the creation of the International Space Station (ISS) [1]. The first ISS modules were launched in the late 1990s. Meanwhile, the ISS enabled the presence of human beings in low Earth orbit for more than 20 years. While ISS will orbit the Earth for a few more years, we will witness exciting new developments driven by space agencies and commercial entities. China will continue to build and utilize its recently established space station. The Artemis program, which is led by the United States National Aeronautics and Space Administration (NASA), will bring human beings back to the moon and may lay the foundation for future human missions to Mars. Finally, commercial providers have begun to offer suborbital and orbital space flights to paying customers. Thus, more and more human beings will experience space travel including individuals who would not qualify for a career as professional astronaut for medical or psychological reasons.

Given its importance in adjusting the cardiovascular system to environmental challenges, the autonomic nervous system has been in the focus of scientists and clinicians involved in human space flight. One of the highlights in this area was an international space shuttle mission dedicated to mechanistic human studies on post-space-flight orthostatic intolerance (Neurolab Mission, STS-90) [2]. The harsh environmental conditions during space travel impose a major burden on the human body including the cardiovascular system [3]. In space, even the healthiest of the healthy will experience worsening of performance and health status unless sufficient countermeasures are instituted. Among more than 30 health risks during long duration space travel, NASA scientists identified five so-called red risks based on their likelihood to occur and their implications for health and performance during and after the mission [4]. These risks comprise consequences of space radiation, isolation and confinement, a hostile and closed environment, the distance from Earth, and altered gravity. Of those, altered gravity elicits particularly strong effects on cardiovascular control through volume redistribution in the body or altered vestibular signaling among other mechanisms. However, all the other environmental challenges could also affect the autonomic nervous system. For example, altered atmosphere conditions in a closed environment could engage peripheral or central chemoreceptors which regulate the autonomic nervous system. In the event that neurons in the brain in autonomic control circuits are being hit by heavy ions, which are important and difficult to shield components of galactic cosmic radiation, grave consequences could occur [5]. Finally, the psychological stresses associated with confinement and isolation could conceivably affect the autonomic nervous system [6].

This review provides an overview on human autonomic research under real and simulated space conditions with a focus on orthostatic intolerance. In addition to enabling safe human space flight, this research may have implications for patients with disorders affecting cardiovascular autonomic control on Earth.

## Orthostatic tolerance—focus on the autonomic nervous system

Environmental challenges are sensed through baroreceptors, chemoreceptors, and the vestibular system among other afferent inputs [7–9]. The information is integrated at the brainstem level and leads to adjustments in efferent sympathetic and parasympathetic activity. Sympathetic stimulation increases heart rate and cardiac contractility, increases vascular tone, and promotes renal sodium reabsorption through renin release and direct tubular actions. Parasympathetic activation primarily decreases heart rate at the level of the sinus node. The importance of the autonomic nervous system in cardiovascular control is illustrated by patients with severe autonomic failure. In these patients, efferent sympathetic and parasympathetic counter-regulation is almost completely lost such that hemodynamic stresses imposed by standing produce profound orthostatic hypotension [10]. Other seemingly trivial hemodynamic challenges such as eating, taking a hot shower, or drinking alcohol massively reduce blood pressure. These stresses are easily compensated for by autonomic counter-regulation in healthy young persons. However, sufficiently strong stimuli, such as exposure to hypergravity on a human centrifuge, in a high-performance aircraft, or during rocket launch or reentry may exceed the compensatory capacity of the healthy autonomic nervous system. Changes in volume status, such as acute blood loss, or cardiovascular deconditioning can also impair tolerance to gravitational challenges [11, 12]. Thus, unusually strong environmental stimuli, impaired hemodynamic reserve, or changes in autonomic nervous system control may elicit cardiovascular symptoms, particularly orthostatic intolerance and syncope [13–15].

The complex interactions between environment and autonomic cardiovascular control that ultimately determine orthostatic tolerance cannot be captured by single autonomic measurements. There is no gold standard approach telling the whole story. Instead, different methodologies and experimental setups have to be combined to elucidate the chain from transduction and sensing of an environmental stressor to autonomically mediated cardiovascular responses.

## Orthostatic intolerance following space travel

Launch and landing of space vessels are associated with major changes in gravitational loading of the human body. During launch, engine thrust accelerates the rocket thereby imposing substantial gravitational forces on human beings. Gravitational force-induced loss of consciousness, known as g-LOC by cognoscenti, could occur. During reentry into the atmosphere and the landing phase, the spacecraft rapidly decelerates which also generates gravitational stress to the body, normally approximately 3 to 5 times the gravity of the Earth. During space shuttle reentry, heart rates exceeding 150 beats/min have been reported. The response likely resulted from combined physiological and psychological stresses [15, 16]. Thanks to gifted space engineers who incorporate human physiology knowledge in their designs, gravitational loading of the cardiovascular system is kept in a safe range except for true emergency situations. Gravitational overloading is avoided through proper positioning of astronauts orthogonally to the acting gravity main vector (chest-to-back direction) during ascent and landing and through planning of flight trajectories among other measures. Even greater challenges to cardiovascular control can occur in other settings, such as in pilots of high-performance aircrafts who are exposed to accelerations in a head-to-foot direction, and are, therefore, not the focus of this review.

**Fig. 1:**
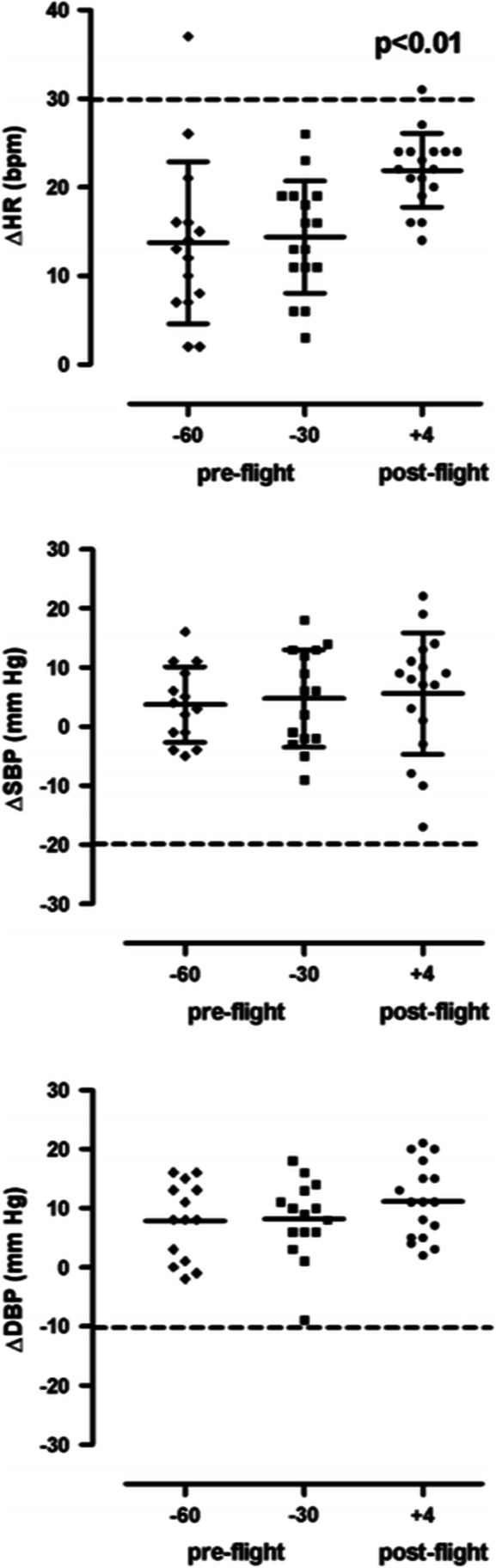
Mean changes in heart rate (∆HR, top), systolic blood pressure (SBP, middle), and diastolic blood pressure (DBP, bottom) in 18 Russian cosmonauts during orthostatic testing at 60 days before space flight (pre-flight, − 60), at 30 days before space flight (− 30 pre-flight), and 3–5 days after space flight (+ 4, post-flight). *p* < 0.01, paired *t* test between pre- and post-flight values (from Tank J. et al. Clin Auton Res 2011;21(2) with permission)

Cardiovascular autonomic symptoms related to exposure to space conditions rather than launch or landing phases have been observed early into human space flight. Physicians taking care of astronauts noted substantial orthostatic intolerance following return to Earth, a syndrome which was coined postflight orthostatic intolerance. It is safe to say that early missions were particularly heroic and stressful to astronauts. In addition to exposure to space conditions like weightlessness, astronauts were under immense psychological and physiological stress. Over the years, medical care improved together with advances in technology and individualized exercise programs. Nowadays, most astronauts are able to stand after several months onboard ISS without presyncope or syncope briefly following return to Earth. Notably, none out of 12 astronauts experienced orthostatic hypotension during the first 24 h after return from 6 months in space when investigated during activities of daily living [17]. Yet, nine out of fourteen astronauts returning from 9–14 days space shuttle missions were unable to complete a 10-min standing test conducted within 4 h after landing [15]. In the upright position, heart rate and systemic vascular resistance were markedly increased while cardiac output and stroke volume were reduced compared with measurements before the mission [15]. Russian cosmonauts returning from several months ISS missions showed a modest increase in upright heart rate within the first days after landing (Fig. 1) [18]. Upright blood pressure was well maintained. Orthostatic symptoms while generally mild-moderate and easy to manage on Earth could pose grave risks when landing on another celestial body.

## Orthostatic intolerance in terrestrial models

Medical research in space is limited by the small number of potential test subjects and difficulties in conducting measurements. Assessments, which are trivial on Earth, such as obtaining and analyzing a blood sample, pose major challenges in space. Parabolic flights can produce weightlessness or reduced g-loads akin to conditions on Mars or the moon for approximately 20–30 s. We have applied the approach to assess the initial orthostatic blood pressure and heart rate responses during different gravity loads [19, 20]. Yet, the short duration of altered gravity and potential confounding through rapid changes in gravity during flight maneuvers are major limitations. Therefore, terrestrial models mimicking certain aspects of space travel have been developed and successfully applied over the years [21].

In cardiovascular autonomic research, bedrest in the head-down tilt position or dry immersion studies have been proven useful. In head-down bedrest studies, participants remain lying with the whole bed tilted head down typically by − 6°, a modest Trendelenburg position. All activities including eating and personal hygiene are conducted in this position. In dry immersion, participants are placed in a water tank with a water-resistant sheet that prevents direct contact with water. Both models unload the cardiovascular system and shift volume towards central circulatory compartments and the head. The fact that horizontal bedrest induces cardiovascular deconditioning and reduces orthostatic tolerance is known for more than 70 years [22]. One study assessed orthostatic responses before and following 6-h bedrest in a − 5° head-down position, which produces cephalad fluid shifts similarly to weightlessness, and in a + 10, + 20, or + 42° head-up position on separate days [23]. Upright heart rate was significantly increased following head-down bedrest and less so after head-up bedrest. In another study, orthostatic tolerance determined by lower body negative pressure (LBNP) decreased within 20 h of − 5° head-down bedrest [24]. The concept of applying negative pressure to the lower part of the body was originally developed for manned space flights in order to counteract the headward fluid shifts and to simulate orthostatic stress in weightlessness. In a 21-day head-down bedrest study, a duration more relevant for a space mission, orthostatic tolerance was measured by combined head-up tilt testing and LBNP and quantified as the time to abortion of the test [25]. On average, orthostatic tolerance was 21 min before and only 12 min following head-down bedrest.

Observations in space and in terrestrial models mimicking space conditions suggest that all three components affecting orthostatic tolerance may be perturbed, namely volume status, cardiovascular fitness, and autonomic nervous system control.

## Changes in volume status

In the early days of human space flight, astronauts may have experienced dehydration and volume loss due to inadequate water and solute supply in the face of space motion sickness, heat stress, and excess sweating. Furthermore, astronauts avoided drinking prior to launch due to the difficulties of voiding in space [26]. However, changes in volume status and volume distribution also occur when water and nutrient supply is optimized as it is today.

Immediately when astronauts enter weightlessness, fluid and blood cells are distributed towards the head. The response is associated with paradoxical central venous pressure reduction [27, 28]. Fluid shifts towards the head and fluid redistribution from the intravascular to the interstitial and intracellular spaces contribute to face reddening and swelling—the so-called puffy face—and nose stuffiness in astronauts [29]. The internal jugular vein is dilated and shows stagnant or even retrograde flow in weightlessness [30], which may predispose to neck vein thrombosis [30, 31]. More chronically, fluid shifts towards the head may contribute to structural changes in the brain and optic disc edema, which are part of the space-flight neuro-ocular syndrome [32–34]. Cerebral magnetic resonance imaging of astronauts after short and long duration space missions depicted upward shift of the brain and white and gray matter volume changes [33, 35]. In another study, an increase in blood markers for brain damage emerged in the first week after return from long duration missions [36]. Mastoid effusions may also occur [37]. Invasive intracranial pressure measurements, which would be unrealistic in astronauts during space missions, have been conducted in patients with Ommaya reservoirs in hyperacute weightlessness during parabolic flights. Ommaya reservoirs are catheter systems placed in cerebral ventricles with a subcutaneous port for infusion of anticancer chemotherapy that can also be used for intracranial pressure measurements. Intracranial pressure during weightlessness differed little from supine intracranial pressure in normal gravity [38]. Possibly, intracranial pressure may not reach pathological levels in space. Yet, on Earth the brain may be protected by daily caudal volume shifts in the upright body position.

**Fig. 2:**
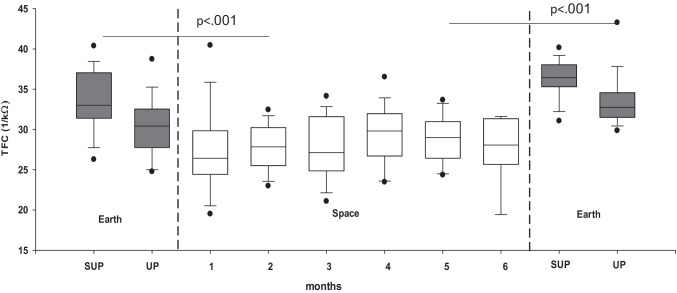
Median thoracic fluid content (TFC) estimated by impedance measurements in 16 Russian cosmonauts before launch in the supine (SUP) and upright (UP) position, monthly onboard the International Space Station, and supine and upright after return to Earth (from Frings-Meuthen P. et al. Circulation 2020;141(19) with permission)

In addition to fluid shifts towards the head, plasma volume, red blood cells, and total blood volume decrease within days in space. [15] The response may be explained in part by fluid shifts from the intravascular to the intracellular compartment [39]. Furthermore, destruction of premature and young mature red blood cells may be an adaptive response to weightlessness [40]. Bioimpedance measurements suggest that there are sustained reductions in thoracic blood volume during long duration space missions (Fig. 2) [41]. Circulating mid-regional pro atrial natriuretic peptide concentrations also decrease, after a transitory 80% increase on the first day [42], in space independently of dietary sodium ingestion [41]. In a short-term − 5° head-down bedrest study, central venous pressure transiently increased followed by normalization over several hours, which differs from the response in space [24]. Yet, cephalad fluid shifts also occur and may lead to ocular changes resembling those observed in astronauts affected by the space-flight-associated neuro-ocular syndrome [43]. Mastoid effusion can also occur during head-down tilt bedrest [44]. Plasma volume decreases during head-down tilt bed rest [45]. Thus, reductions in plasma volume in weightlessness and in head-down bed rest likely put an additional strain on orthostatic tolerance.

## Cardiovascular deconditioning

Deconditioning is the expected response of the cardiovascular system to unloading be it in weightlessness or during bedrest. In astronauts, cardiopulmonary fitness was maintained after approximately 2 weeks in space but was reduced directly after return to Earth [46]. Following approximately 2-week horizontal bedrest, healthy women and men showed a marked reduction in maximal oxygen uptake [47]. It is difficult, however, to discern primary changes in cardiovascular structure and function from secondary influences of volume status and autonomic regulation on cardiopulmonary performance and orthostatic tolerance [48, 49]. Nevertheless, changes in cardiovascular structure and function may occur following space flight and in terrestrial models. In particular, reductions in left ventricular mass have been observed in astronauts following space flight and following 12 weeks horizontal bedrest [50]. However, the response of left ventricular mass to bedrest deconditioning may be heterogenous [49, 51]. One study showed reversal of cardiac atrophy within days after return from space, which may suggest that the response was mediated by fluid shifts rather than true cardiac atrophy [52]. A small heart together with reductions in volume status could contribute to orthostatic symptoms as evidenced by patients with the postural tachycardia syndrome [14, 53]. Influences of real and simulated space conditions on vascular structure and function have been recently reviewed [54]. Whether vascular changes, either on the venous or on the arterial side, contribute to orthostatic intolerance is unclear. The interpretation of the literature is complicated by the fact that in space, some aspects of cardiovascular deconditioning are addressed more than others. For example, astronauts regularly conduct endurance and strength training to maintain exercise capacity. Yet, there is no adequate substitute for terrestrial gravity challenges to the cardiovascular system.

## Sympathetic nervous system adaptation

Blood pressure is maintained during orthostatic stress when sympathetic actions on heart, blood vessels, and kidney are sufficiently increased. The response requires that post-ganglionic sympathetic neurons are activated and release norepinephrine, which then engages postganglionic adrenergic receptors in cardiovascular organs or the kidney. The response is primarily terminated through neuronal re-uptake of released norepinephrine. Therefore, changes in norepinephrine release, responsiveness, or uptake could affect orthostatic tolerance following space flight.

In astronauts who underwent muscle sympathetic nerve activity measurements through microneurography, sympathetic activity was increased after space flight, both, while supine and during head-up tilt [55]. The response was proportional to reductions in cardiac stroke volume postflight. Inflight measurements of muscle sympathetic activity and norepinephrine spillover during a space shuttle mission, a remarkable accomplishment considering the complexity of these methods, showed modest increases in sympathetic neural traffic and norepinephrine release [56]. However, during parabolic flight in seated healthy men and women, muscle sympathetic nerve activity was 191% during hypergravity but only 82.8% during weightlessness compared with normal gravity [57]. In two Russian cosmonauts who underwent multiple plasma and urinary determinations before, during, and after a long-term space mission, catecholamine measurements did not show major changes in space compared to before the mission. However, these measurements increased sharply in the days following the mission [58]. An increase in plasma norepinephrine following space flight was also shown in another study [59]. Yet, eight male astronauts showed no changes in plasma noradrenaline and adrenaline concentrations during and after their long duration ISS missions with respect to preflight values, despite a 35–41% increase in cardiac output and an 8–10 mmHg decrease of arterial pressure in space [60].

Among 40 astronauts who had finished space missions of up to 16 days, those who were unable to remain standing for 10 min showed a markedly reduced increase in venous plasma norepinephrine compared to those who were able to remain standing. The group with less orthostatic tolerance also exhibited lower standing systemic vascular resistance. Women may be more likely to show such a response [61]. Astronauts with less orthostatic tolerance showed markedly raised dihydroxyphenylglycol (DHPG) plasma concentrations along with an attenuated norepinephrine response [62]. Because, norepinephrine is converted to DHPG through monoamine-oxidases following re-uptake from the synaptic cleft, the finding may indicate an increase in norepinephrine uptake and metabolism. An increase in tyramine-mediated norepinephrine release supports the idea [62]. Overall, it appears unlikely that space conditions render the sympathetic nervous system unable to respond to orthostatic stress, Yet, individual differences in sympathetic responsiveness may make an astronaut less or more likely to experience orthostatic intolerance following space flight.

Following head-down bedrest studies, participants showed unchanged or modestly increased resting sympathetic nerve traffic [63–65]. The sympathetic response to handgrip exercise was unchanged [65]. The reduction in blood pressure during phase 2 of the Valsalva maneuver was exacerbated by head-down bedrest; however, sympathetic activity increased appropriately [66]. A more detailed multiunit action potential analysis of sympathetic nerve recordings suggested that head-down bedrest may induce changes in neural recruitment strategies, particularly during breath holding [64]. Despite a reduction in plasma volume over time, urinary norepinephrine was substantially reduced, and plasma norepinephrine was largely unchanged towards the end of bedrest [67]. Another head-down bedrest study showed a similar response [68]. The authors reasoned that an inappropriate sympathetic response to hypovolemia could predispose to orthostatic intolerance following bedrest. The idea is supported by the observation that persons with orthostatic intolerance following head-down bedrest showed a blunted increase in sympathetic nerve traffic with standing and insufficient vasoconstriction in the splanchnic circulation and in the legs [65, 69].

## Parasympathetic heart rate control

Vagal withdrawal is the first response when assuming the upright position. However, patients with a denervated heart, such as heart transplant recipients, do not show major orthostatic symptoms. Moreover, cardiac pacemakers while attenuating bradycardia during tilt table testing are of limited utility in improving orthostatic tolerance of patients with vasovagal syncope [70]. Similarly, intravenous atropine did not prevent syncope during head-up tilt testing in healthy men [71]. On the other hand, patients with sympathetic vasomotor lesions faint during orthostatic challenges despite a substantial increase in heart rate [72]. However, pharmacological norepinephrine transporter blockade, which primarily raises heart rate, prevented presyncope during head-up tilt testing in healthy persons [73]. Overall, influences of heart rate on orthostatic tolerance are limited.

Nevertheless, altered parasympathetic activity might modify orthostatic heart rate responses following space flight. Compared with before space flight, heart rate was higher, and heart rate variability was reduced on landing day [59]. Heart rate variability was well maintained in Russian cosmonauts during long duration missions. During sleep, cosmonauts onboard the MIR station showed reductions in heart rate together with an increase in heart rate variability in the high frequency range, which primarily results from parasympathetic influences on the sinus node [74]. Another study showed an opposite heart rate variability response during sleep [75]. However, maximal heart rate variability during deep breathing declined in six out of seven cosmonauts suggesting that there may be reduction in vagal reserve [76]. Following return to Earth, astronauts who were unable to complete a standing test showed an increase in heart rate variability while supine [77].

## Autonomic reflex regulation

As outlined above, parasympathetic and sympathetic efferent nerves are controlled by brain stem nuclei that receive input from various afferents and higher brain areas, especially from the insula [78]. Therefore, changes in these reflex circuits could contribute to altered autonomic cardiovascular control in real or in simulated space conditions. Baroreflex and vestibular mechanisms are prime suspects. The relevance of reflexes of the low-pressure system, e.g., the heart rate response to an acute increase in blood volume (Bainbridge reflex), is unclear.

Baroreflex-mediated heart rate responses elicited through neck suction were attenuated during and after short-term space flight [79]. Another study using transfer function analysis showed reduced low-frequency gain of the baroreflex after flight [80, 81]. Repeated baroreflex sensitivity measurements using the sequence and spectral alpha techniques in four astronauts during a 16-day flight showed an initial increase in baroreflex-mediated heart rate control returning to baseline values at the end of space flight [81]. Interestingly, vagal heart rate control was reduced in 5 European but not in 5 Chinese astronauts, which raises questions regarding genetic variation in the response to space conditions [82]. In contrast, sympathetic responses to different stimuli like the Valsalva maneuver, head-up tilt, or lower body negative pressure seem to be enhanced during and after space flight [55, 56, 83]. Data about changes on autonomic reflex regulation from long-term missions is still lacking.

The central shift of body fluids after transition into weightlessness should, in theory, activate volume receptors located in central veins, pulmonary arteries, and cardiac atria with subsequent vagal withdrawal and heart rate increases [84]. However, Chinese astronauts showed heart rate reductions in space [85]. Possibly, low-pressure reflexes are masked by a dominant arterial baroreflex.

The vestibular system, which regulates autonomic efferents [86], is profoundly perturbed in weightlessness [9, 87]. Long-term space missions may attenuate vestibular influences on the sympathetic nervous system and, thereby, negatively affect orthostatic tolerance [88, 89]. Space flight may also affect the coupling between breathing and efferent autonomic activity, which is of unclear significance for orthostatic tolerance [90].

## Is cerebral autoregulation perturbed after space flight?

Theoretically, an impairment in cerebral autoregulation, which maintains blood flow over a wide range in blood pressure values, could compromise orthostatic tolerance following space flight. The topic including the different methodological approaches to determine static and dynamic cerebral autoregulation has been recently reviewed [91, 92]. Remarkably, cerebral vasoconstriction and hypoperfusion with hyperacute gravity transitions during parabolic flights may predict poor orthostatic tolerance [93].

In six astronauts participating in a space shuttle mission, dynamic cerebral autoregulation was determined before, during, and after space flight at rest and during orthostatic stress. If anything, cerebral autoregulation was improved after the mission [94]. During more long-term missions, cerebral blood flow velocity increased in proportion to a reduction in hemoglobin levels [95]. The authors suggested that increased flow may have compensated reductions in blood oxygen carrying capacity. Cerebral blood flow velocities during incremental lower body negative pressure testing were lower after compared to before a head-down bedrest study [96]. While subtle changes in cerebral autoregulation may occur, particularly during more long-term missions, there is no evidence for a consistent and profound impairment.

## Potential orthostatic intolerance countermeasures for space flight

Since NASA plans long duration human missions with up to 1100 days in space, development of countermeasures maintaining astronauts’ health and performance is an important goal. Given the importance of volume regulation in the pathogenesis of postflight orthostatic intolerance, interventions affecting fluid balance or sodium homeostasis appear sensible. In a bedrest study in which plasma volume was restored to pre-bedrest levels through intravenous isotonic fluids and oral salt, presyncope or syncope after bedrest was improved [97]. However, volume loading did not prevent orthostatic tachycardia. In a 7-day head-down bedrest study, treatment with the mineralocorticoid fludrocortisone over 24 h was more effective in maintaining plasma volume and orthostatic tolerance compared to oral salt and water loading [98]. Remarkably, baroreflex heart rate regulation was also better maintained in the fludrocortisone group. However, only seven persons per group were investigated. In a study conducted in space shuttle astronauts, one group received usual care, while another group increased fluid and salt intake before return to Earth. Fluid and volume loading substantially attenuated standing heart rate and stabilized blood pressure with standing [99]. Among astronauts who were treated with either a single fludrocortisone dose or placebo 7 h prior to landing, those receiving fludrocortisone did not show an obvious improvement in orthostatic tolerance [100].

Rowing exercises preserved cardiopulmonary fitness and orthostatic tolerance during a head-down tilt bedrest study [101].

In a case report, a female astronaut with post-flight orthostatic intolerance exhibited an improved orthostatic response following a subsequent space flight after ingesting a single dose of the alpha-adrenoreceptor agonist midodrine [102].

Anti-G suits as well as garment systems for the calf, thigh, and splanchnic areas were tested effective in preventing acute volume shifts and increase orthostatic tolerance after real and simulated gravity [103–107].

Countermeasures mimicking some or all aspects of standing on Earth while being in space have been actively investigated over many years. Thigh cuffs reducing venous drainage applied over several hours per day, an approach introduced by Russian cosmonauts [108], did not improve orthostatic tolerance following head-down bedrest [109]. However, the intervention may have had a beneficial effect on plasma volume and heart rate control. Whether lower body negative pressure training towards the end of space flight is effective in restoring orthostatic tolerance is questionable; however, cardiovascular responses to the intervention predict cardiovascular responses after returning to Earth [110]. Artificial gravity generated through a short arm centrifuge could be beneficial in attenuating the physiological deterioration in weightlessness [111]. Yet, artificial gravity alone may not suffice [49].Combinations of artificial gravity and exercise training hold promise [112]. During the Neurolab mission, four out of six crew members were exposed to 1 g on a centrifuge installed inside the Space Shuttle Columbia. After return to Earth, none of the crew members in the centrifugation group experienced orthostatic intolerance, whereas one of the two crew members in the control group showed such symptoms [113].

## Applications on Earth

Observations in astronauts remind us that the ability of the autonomic nervous system to cope with terrestrial gravity cannot be taken for granted. It is no surprise that orthostatic intolerance, which comes in different expressions, is a common clinical challenge in earthlings [114, 115]. Roughly, orthostatic intolerance syndromes can be divided in neurally mediated syncope, postural tachycardia syndrome (POTS), and orthostatic hypotension. Neurally mediated syncope is among the most common reasons for emergency room visits [116]. Patients with neurally mediated syncope have perfectly normal cardiovascular autonomic control until a trigger, such as prolonged standing, sets off hypotension with or without bradycardia. Owing to its name, POTS is characterized by an excessive heart rate response and hyperadrenergic symptoms with standing [117]. POTS is among the most common autonomic nervous system disorders [118] and primarily but not exclusively affects younger women. Orthostatic hypotension, which is characterized by sustained reductions in blood pressure with standing, becomes more prevalent with increasing age [10]. Milder form of orthostatic hypotension, which is often explained by multiple factors, such as age-associated declines in autonomic regulation, deconditioning, and medications among others, is rather common in older people. Profound orthostatic hypotension usually points towards a severe underlying condition, such as multiple system atrophy, pure autonomic failure, or autoimmune autonomic ganglionopathy to name a few.

Phenotypically, orthostatic intolerance following real or simulated space flight resembles neurally mediated syncope or POTS rather than the immediate and then sustained reduction in blood pressure seen in patients with orthostatic hypotension. It appears that orthostatic intolerance following space flight is not explained by a single mechanism. Ultimately, individual predisposition, volume loss, cardiovascular deconditioning, autonomic nervous system adaptation, and, perhaps, changes in cerebral autoregulation may have an additive negative effect on orthostatic tolerance. While all the orthostatic intolerance syndromes that we encounter in patients in the clinic have different underlying causes, the observation that multiple factors determine orthostatic tolerance is clinically relevant. For example, hypovolemia makes matters worse in all these conditions. Conversely, measures attenuating venous pooling may improve neurally mediated syncope, POTS, and orthostatic hypotension. Yet, targeting a single mechanism rarely suffices in severely affected patients. For example, simply starting a patient with severe orthostatic hypotension on pressor drugs rarely controls symptoms unless other treatments, such as dietary salt intake, water ingestion, compression garments, and so on are instituted [10]. However, in severely affected patients, currently available therapies may not suffice in improving symptoms. Novel therapies such as spinal cord pacing [119] or deep brain stimulation [120] may have utility in this setting. We certainly hope that the engineering expertise, which is constantly pushed to its limit in space research, will yield new treatments for our patients with orthostatic intolerance on Earth.
